# Clinical Efficacy of Thoracoscopic Surgery with the da Vinci Surgical System versus Video-Assisted Thoracoscopic Surgery for Lung Cancer

**DOI:** 10.1155/2022/5496872

**Published:** 2022-06-08

**Authors:** Jin-Cai Zhou, Wu-Ping Wang, Shu-Qiang Wu, Jia-Lin Wang, Wen-Hai Li

**Affiliations:** ^1^Department of Thoracic Surgery, Xi'an International Medical Center Hospital, Xi'an, China; ^2^Department of Chest/Breast Surgery, Xi'an Fengcheng Hospital, Xi'an, China

## Abstract

**Objective:**

To assess the clinical efficacy of thoracoscopic surgery with the da Vinci surgical system versus video-assisted thoracoscopic surgery (VATS) for lung cancer.

**Methods:**

From August 2019 to December 2020, 193 patients with lung cancer assessed for eligibility scheduled for surgery in our hospital were recruited and assigned at a ratio of 1 : 1 to receive VATS (control group) or thoracoscopic surgery with the da Vinci surgical system (research group). The primary measurement is the clinical efficacy of the two surgical modalities.

**Results:**

The baseline features of the research group were comparable with those of the control group (*P* > 0.05). Besides, the two groups showed similar tumor types, tumor locations, and clinicopathological staging (*P* > 0.05). Da Vinci surgical system-assisted thoracoscopic surgery had short operative time, less intraoperative blood loss, better lymph node dissection, and lower intraoperative conversion rates compared to VATS. Compared with the control group, the research group had significantly higher postoperative forced expiratory volume in one second (FEV1), forced vital capacity (FVC), maximal voluntary ventilation (MVV), the functional assessment of cancer therapy-general module (FACT-G) of the FACT-lung (FACT-L) Chinese version V4.0, lung cancer-specific module scores, and total scores (*P* < 0.05). The research group showed better postoperative drainage volume, shorter intubation duration, and length of hospital stay and a lower incidence of complications versus the control group (*P* < 0.05). The da Vinci surgical system reduced the probability of intraoperative mistakes and better ensured a safe and satisfactory surgery.

**Conclusion:**

The thoracoscopic surgery with the da Vinci surgical system better reduces intraoperative and postoperative bleeding, shortens drainage and intubation duration, enhances the lung function and survival quality of patients, and lowers the risk of surgical mistakes to ensure surgical safety versus VATS.

## 1. Introduction 

Lung cancer is the most common malignancy with the highest incidence and mortality rate globally, with an estimated 2 million new cases and 1.76 million deaths per year [1]. The incidence and mortality of lung cancer have markedly increased across many countries in the past five decades, and the etiology of lung cancer is still poorly understood [2]. Surgical resection is the mainstay of treatment for lung cancer, and video-assisted thoracoscopic surgery (VATS) is clinically [3] used to effectively minimize incision trauma with well-recognized effectiveness and safety; however, its long-term prognosis remains similar to that of traditional open-heart surgery [4]. The da Vinci surgical system is designed to perform complex surgical procedures through the use of a minimally invasive approach, which was first applied to lung surgery by Melfi et al. in 2002 [5]. Robotic-assisted thoracoscopic surgery (RATS) using the da Vinci surgical system provides an intuitive and clear field of view, magnifies the tissues in the operative field up to 10–15 times, and provides panoramic 3D images that are true 16 : 9 scale images, with advantages unavailable in 2D images of thoracoscopy [6]. RATS substantially alleviates surgeon fatigue during surgery and enhances concentration, and the flexibility and precision of the machine and the absence of tremors ensure the accuracy of the procedure [7]. In recent years, it has been widely used in various disciplines at home and abroad, and research reports from several centers in China have confirmed that da Vinci robotic-assisted thoracoscopic surgery is a safe and effective new procedure with the advantages of less intraoperative bleeding, faster postoperative recovery, and fewer complications [8, 9]. Accordingly, 193 patients with lung cancer assessed for eligibility scheduled for surgery in our institution were recruited between August 2019 and December 2020 to assess the clinical efficacy of thoracoscopic surgery with the da Vinci surgical system versus VATS for lung cancer and to provide a clinical reference basis for better treatment selection. The results are as follows.

## 2. Materials and Methods

### 2.1. Baseline Data

From August 2019 to December 2020, 193 patients with lung cancer assessed for eligibility scheduled for surgery in our hospital were recruited and assigned to receive VATS (control group, *n* = 87) or thoracoscopic surgery with the da Vinci surgical system (research group, *n* = 86). The baseline features of the research group (58 males and 29 females, mean age of (48.12 ± 8.37) years, and BMI of (24.19 ± 2.35) kg/m^2^) were comparable with those of the control group (47 males and 39 females, mean age of (49.36 ± 7.18) years, and BMI of (24.06 ± 1.97) kg/m^2^) (*P* < 0.05) (Table 1). Patients' data on tumor types, tumor locations, and clinicopathological staging were collected.

### 2.2. Inclusion and Exclusion Criteria

Inclusion criteria were as follows: patients aged ≥ 18 years old; patients with pathological results of lung cancer; and patients with no recent treatment with other drugs. Exclusion criteria were as follows: patients with missing or incomplete clinical data; patients with serious organ diseases such as the heart, liver, and spleen; and patients with surgery-related contraindications.

All patients and their families were informed of this study and provided written informed consent. This study was approved by the Ethics Committee of the Xi'an Fengcheng Hospital, no. K77903.

## 3. Methods

The patients in the control group received VATS. The observation port of approximately 1.5 cm was made at the 7-8th intercostal space in the midaxillary line, the main operation hole of 3-4 cm was made at the 3-4th intercostal space in the anterior axillary line, and the secondary operation hole of 2 cm was made at the 6-7th intercostal space in the posterior axillary line. The pulmonary vessels and bronchi were treated sequentially, and the diseased lung lobes, systemic hilum, and mediastinal lymph nodes were resected, followed by the closure of the chest after placement of a drainage tube.

The patients in the research group underwent thoracoscopic surgery with the da Vinci surgical system. The light source hole was selected at the 9th intercostal space in the midaxillary line, and the 1st and 2nd arm operation holes were placed, opposite the lung hilum, and the incision position was adjusted according to the patient's body size and tumor location. The lens and robot arm were switched and adjusted to perform sequential treatment on the lung arteries, veins, and bronchi to resect the diseased lung lobes, and after systematic dissection of the hilar and mediastinal lymph nodes, the chest was closed with the placement of a drainage tube.

### 3.1. Outcome Measures

1. The operative time, intraoperative blood loss, lymph nodes dissection, and intraoperative conversion rates of the two groups were collected and compared
2. Pulmonary function tests, including forced expiratory volume in one second (FEV1), forced vital capacity (FVC), and maximal voluntary ventilation (MVV), were performed 1 month after surgery to compare and analyze the pulmonary function of the two groups of patients
3. The Chinese version of functional assessment of cancer therapy-lung (FACT-L) (V4.0) was used to measure the survival quality of patients, including the FACT-general module (FACT-G) and the lung cancer-specific module, with a total of 36 entries, all using a 5-point scale of 0–4 points. The higher the total score, the better the survival quality.
4. The postoperative drainage volume, postoperative hospital stay, and complications in both groups were recorded and analyzed. The complications included the pleural infection, intrathoracic hemorrhage, bronchopleural fistula, respiratory failure, and respiratory complications.
5. The specific operation modalities in surgery, including hand-eye coordination, surgical operation control, imaging situation, and flexibility, were compared

### 3.2. Statistical Analysis

SPSS 22.0 software was used for data analysis, and GraphPad Prism 8 software was used for image rendering. The count data were expressed as (*n* (%)) and was analyzed using the chi-square test. The measurement data were expressed as ($\bar{\text{x}}$ ± *s*) and were processed by the *t*-test. Differences were considered statistically significant at *P* < 0.05.

## 4. Results

### 4.1. Clinical Data

**Figure 1:**
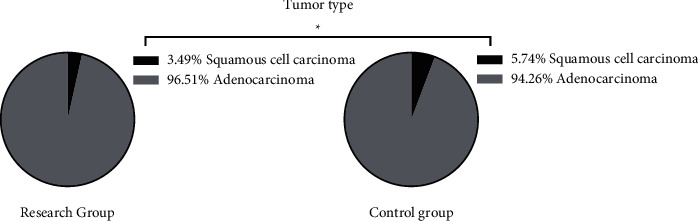
Tumor types of the two groups (%). ^*∗*^*P* > 0.05 in comparison of tumor types between the two groups.

**Figure 2:**
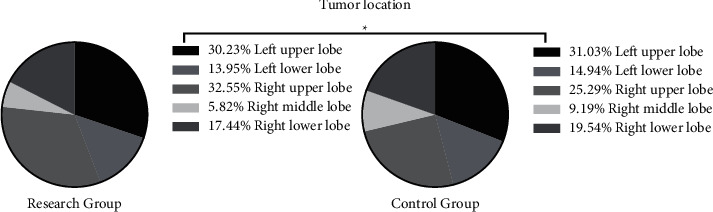
Tumor location of the two groups (%). ^*∗*^*P* > 0.05 in comparison of tumor location between the two groups.

**Figure 3:**
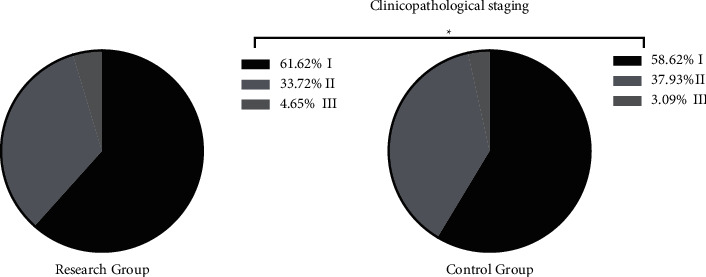
Clinicopathological staging of the two groups (%). ^*∗*^*P* > 0.05 in comparison of clinicopathological staging between the two groups.

The two groups showed similar tumor types, tumor locations, and clinicopathological staging (*P* > 0.05) (Figures 1, 2, and 3).

### 4.2. Intraoperative Conditions

Da Vinci surgical system-assisted thoracoscopic surgery outperformed VATS in terms of operative time (125.61 ± 35.79 vs. 139.44 ± 33.28), intraoperative blood loss (88.65 ± 35.17 vs. 103.45 ± 28.94), lymph node dissection (7.23 ± 1.23 vs. 8.95 ± 1.77), and intraoperative conversion rates (2.32% vs. 6.89%) (*P* < 0.05) (Table 2).

### 4.3. Lung Function

Significantly higher postoperative FEV1 (3.25 ± 0.71 vs. 2.54 ± 0.82), FVC (3.71 ± 0.5 vs. 3.12 ± 0.67), and MVV (55.84 ± 5.49 vs. 52.14 ± 5.03) were found in the research group versus the control group (*P* < 0.05) (Table 3).

### 4.4. Quality of Life

At one month after surgery, the FACT-G module score, lung cancer-specific module score, and total score in the Chinese version of FACT-L (V4.0) of the study group patients (89.92 ± 9.11, 18.87 ± 3.24, and 109.08 ± 11.64) were significantly higher than those in the control group (80.23 ± 12.38, 18.01 ± 2.13, and 100.36 ± 13.81) (*P* < 0.05) (Table 4)

### 4.5. Postoperative Conditions

The research group showed better postoperative drainage volume (1.89 ± 1.84 vs. 1.17 ± 1.05), shorter intubation duration (4.97 ± 1.56 vs. 4.01 ± 1.02), and length of hospital stay (7.25 ± 2.36 vs. 6.15 ± 1.21) and a lower incidence of complications (4.59% vs. 1.16%) versus the control group *P* < 0.05) (Table 5).

### 4.6. Surgical Operations

The thoracoscopic surgery with the da Vinci surgical system features more advantages and can be performed by one surgeon. It can magnify the field of view 10–15 times and provide clear 3D stereoscopic images, with the precise control of the robotic arm to lower the probability of surgical mistakes and better ensure the safety and smooth completion of the operation (Table 6).

## 5. Discussion

Lung cancer is a malignant tumor originating from the bronchial mucosa or glands of the lung and usually shows a complex clinical presentation with symptoms such as cough, wheezing, chest pain, hemoptysis, or blood in the sputum. It is considered one of the fastest-growing malignancies in terms of morbidity and mortality and one of the most threatening to the life and health of the population [10]. Surgery is the optimal treatment option for lung cancer with favorable treatment efficacy for early stage lung cancer. VATS is clinically used to effectively minimize incision trauma with well-recognized effectiveness, safety, and faster recovery [11, 12]. Nonetheless, the surgery is extremely demanding for the surgeon, and its long-term prognosis remains comparable to that of VATS. Thus, there exists an urgent need to explore more refined, optimized, and minimally invasive surgical options [13]. The da Vinci surgical system is a new generation of robotic surgery systems developed at the beginning of the 21st century [14], which has been applied in many fields such as thoracic surgery, urology, and general surgery successively, and provided satisfactory treatment efficiency [15, 16]. RATS features a sophisticated system and convenient operating functions, provides a clear surgical field of view, and substantially reduces surgeon fatigue during surgery and the risk of intraoperative mistakes, thereby safeguarding the patients' safety [17, 18]. Studies reported in several centers in China have confirmed that da Vinci robotic-assisted thoracoscopic surgery is a safe and effective new procedure with the advantages of less intraoperative bleeding, rapid postoperative recovery, and fewer complications [19, 20]. Of note, the high costs of purchase, maintenance, and consumables are a concern and continue to limit uptake of robot systems in thoracic surgery [21].

In the present study, the better results of intraoperative and postoperative conditions of patients indicated that the da Vinci surgical system shortens the operative time and hospital stay, reduces postoperative drainage volume, lowers the risk of complications, and features a high safety profile. Moreover, the higher lung function scores and quality of life scores from the patients receiving da Vinci robotic-assisted thoracoscopic surgery were attributed to less trauma to the patient's chest wall and less damage to the respiratory muscles by the robotic surgical system. Furthermore, da Vinci robotic-assisted thoracoscopic surgery substantially alleviates surgeon fatigue during surgery and enhances concentration, and the flexibility and precision of the machine and the absence of tremors ensure the accuracy of the procedure.

To sum up, the thoracoscopic surgery with the da Vinci surgical system better reduces intraoperative and postoperative bleeding, shortens drainage and intubation duration, enhances the lung function and survival quality of patients, and lowers the risk of surgical mistakes to ensure surgical safety versus VATS.

## Figures and Tables

**Table 1:** Comparison of baseline data of the eligible patients ($\bar{\text{x}}$ ± *s*).

Groups	*n*	Gender		Mean age	BMI
		Male	Female		
Research group	86	47	39	49.36 ± 7.18	24.06 ± 1.97
Control group	87	58	29	48.12 ± 8.37	24.19 ± 2.35
*t*	—	—	—	1.045	0.394
*P*	—	—	—	0.297	0.694

**Table 2:** Comparison of intraoperative conditions ($\bar{\text{x}}$ ± *s*, %).

Groups	Operative time (min)	Intraoperative blood loss (mL)	Lymph node dissection	Intraoperative conversion rates (%)
Research group (*n* = 86)	125.61 ± 35.79	88.65 ± 35.17	7.23 ± 1.23	2 (2.32)
Control group (*n* = 87)	139.44 ± 33.28	103.45 ± 28.94	8.95 ± 1.77	6 (6.89)
*t*/*x*^2^	2.632	3.024	7.414	3.150
*P*	0.009	0.003	<0.001	0.046

**Table 3:** Comparison of lung function ($\bar{\text{x}}$ ± *s*).

Groups	FEV1 (L)	FVC (L)	MVV (L/m)
Research group (*n* = 86)	3.25 ± 0.71	3.71 ± 0.58	55.84 ± 5.49
Control group (*n* = 87)	2.54 ± 0.82	3.12 ± 0.67	52.14 ± 5.03
*t*	6.085	6.189	4.623
*P*	<0.001	<0.001	<0.001

**Table 4:** Comparison of postoperative quality of life ($\bar{\text{x}}$ ± *s*).

Groups	FACT-G	Lung cancer-specific module	Total score
Research group (*n* = 86)	89.92 ± 9.11	18.87 ± 3.24	109.08 ± 11.64
Control group (*n* = 87)	80.23 ± 12.38	18.01 ± 2.13	100.36 ± 13.81
*t*	5.858	2.065	4.488
*P*	<0.001	0.040	<0.001

**Table 5:** Comparison of postoperative conditions ($\bar{\text{x}}$ ± *s*, %).

Groups	Postoperative drainage volume (L)	Postoperative intubation duration (d)	Incidence of complications (%)	Postoperative length of hospital stay (d)
Research group (*n* = 86)	1.17 ± 1.05	4.01 ± 1.02	1 (1.16)	6.15 ± 1.21
Control group (*n* = 87)	1.89 ± 1.84	4.97 ± 1.56	4 (4.59)	7.25 ± 2.36
*t*/*x*^2^	3.156	4.785	4.646	3.851
*P*	0.002	<0.001	0.031	<0.001

**Table 6:** Comparison of surgical operations ($\bar{\text{x}}$ ± *s*, %).

Items groups	Research group	Control group
Image and operation direction	Not in the same direction	In the same direction
Surgical operation control	Requires two-person pairing control	One person can perform the operation
Imaging	Magnify 3–5 times, 2D plane	Magnify 10–15 times, 3D
Flexibility	No articulations, 4 directions of adjustments	Simulated wrist robot with 7 directions of adjustments
Operational precision	Control with hands	Control with robotic arms
Stability	Natural trembling of the human hand	No trembling
Surgical posture	Standing, low comfort	Sitting, high comfort

## Data Availability

The data generated or analyzed during this study are included within the article.
